# Ovariole Structure of the Cochineal Scale Insect, *Dactylophis coccus*

**DOI:** 10.1673/031.008.2001

**Published:** 2008-03-13

**Authors:** A. Ramírez-Cruz, C. Llanderal-Cázares, R. Racotta

**Affiliations:** ^1^ Departamento de Morfología. Escuela Nacional de Ciencias Biológicas. Instituto Politécnico Nacional. Prolongación de Carpio y Plan de Ayala s/n. Col. Casco de Santo Tomás. C.P. 11340. México, D.F. México; ^2^ Entomología y Acarología. *Campus* Montecillo. Colegio de Postgraduados. C.P. 56230. Montecillo, Texcoco, Estado de México. México; ^3^ Departamento de Fisiología. Escuela Nacional de Ciencias Biológicas. Instituto Politécnico Nacional. Prolongación de Carpio y Plan de Ayala s/n. Col. Casco de Santo Tomás. C.P. 11340. México, D.F. México

**Keywords:** germarium, nurse cells, oocyte, trophic cord, vitellarium

## Abstract

The ovaries of the adult cochineal scale insect, *Dactylopius coccus* Costa (Hemiptera: Coccoidea: Dactylopiidae) are made up of more than 400 short ovarioles of the telotrophic type. The ovarioles develop asynchronously. The ovarioles consist of a germarium with six or seven nurse cells, a vitellarium with an oocyte, and pedicel. A terminal filament is lacking. A maturing oocyte was attached to the trophic core by the trophic cord during previtellogenesis and most of vitellogenesis.

## Introduction

The *Dactylopiidae* form a group of phytophagous insects some of which have economic importance. Their specific hosts are plants of the Cactaceae family, mainly the genera *Opuntia* and *Nopalea* (Perez Guerra and Kosztarab 1992). This family includes only the genus *Dactylopius,* which is currently is represented worldwide by 9 species: D. *austrinus* De Lotto, 1974; *D. ceylonicus* (Green, 1986); D. coccus Costa, 1835; *D. confertus* De Lotto, 1974; *D. confusus* (Cockerell, 1893); *D. opuntiae* (Cockerell, 1896); *D. salmianus* De Lotto, 1974; *D. tomentosus* (Lamark, 1801); *D. zimmermannii* De Lotto, 1974 (Perez Guerra and Kosztarab 1992).

D. coccus is the most important species of this family due to its being used for the extraction of carmine acid, a natural red dye presently used in food, pharmaceutical and cosmetic industries, among others (Vigueras and Portillo 2001). As a result of the commercial importance of this insect it has been well studied. Aspects of the biology and behavior (Méndez-Gallegos et al. 1993; Llanderal and Nieto 2001), phylogenic relations (Portillo 2005) and production and exploitation methods of the dye (Marin and Cisneros 1983; Flores-Flores and Tekelenburg 1995; Aldama-Aguilera et al. 2005) have been studied. Cortés et al. (2005) suggested that the reproductive tract of *D. coccus* is a possible site of carmine acid synthesis. Detailed information about the structure of the reproductive system of *D. coccus* females does not exist, despite the importance of this subject for the management of the insect.

The objective of this study was to determine the general histological characteristics of the ovarioles of *D. coccus,* with the purpose of contributing to the knowledge of the reproductive biology of this species, which is of great economic importance.

## Materials and Methods

The adults of *D. coccus* were obtained through cladode infestation of *Opuntia ficus-indica* (L.) var. atlixco (Cactaceae), a host of *D. coccus,* were maintained in 0.50 × 0.26 × 0.30 m glass cases. The cladodes were kept vertical by raffia strings inside the cases, at 6 cm distance from each other. Cultures of D. coccus were maintained on the cladodes of the cactus. Adult females were collected between 5 and 10 days after eclosion. Reproductive tracts were dissected in Ringer solution (Martínez 1999).

For anatomical examination, some reproductive tracts were fixed in 2% glutaraldehyde and post-fixed with 1% osmium tetroxide for observation using scanning electron microscopy (Vázquez and Echeverría 2000). To count the number of nurse cells within the germarium, whole reproductive tracts were fixed in Carnoy's and processed using the Feulgen—green light technique (Martínez 1999). For the description of structural characteristics, the ovaries of females were fixed in aqueous Bouin's, embedded in paraffin and 6 µm thick sections were cut. These sections were then stained using the hematoxylin-eosin technique.

## Results and Discussion

### Female Reproductive System

**Figure 1.**
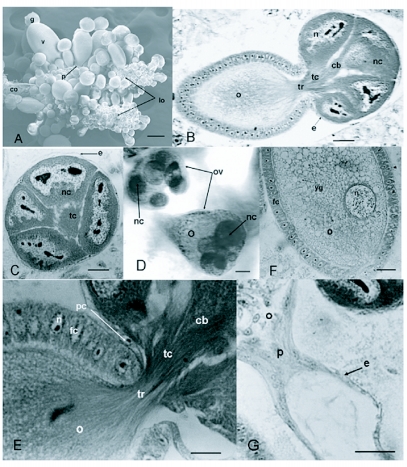
Micrographs of reproductive system of adult female of *Dactylopius coccus.* A, complete reproductive system of adult female. B, longitudinal section through the ovariole. C, transversal section through of germarium. D, complete immature ovarioles. E, longitudinal section through the ovariole. F, longitudinal section through the germarium. G, longitudinal section through the pedicel, cb, trophic processes; co, common oviduct; e, epithelial sheath; fc, follicular cell; g, germarium; lo, lateral oviduct; n, nucleus; nc, nurse cell; o, oocyte; ov, ovariole; p, pedicel; pc, prefollicular cell; tc, trofic core; tr, trophic cord ; v, vitellarium; yg, yolk globules. Scale bars: A = 100 µm; B, C, F, G = 50 µm; D = 5 µm; E = 30 µm.

The reproductive system of the adult *D. coccus* female (Figure 1A) has a pair of ovaries, which are made up of about 400 short telotrophic ovarioles. The number of ovarioles present in Coccoidea is extremely variable, for example, there are around 30 in *Orthezia urticae* (Orthezidae) (Vogelgesang and Szklarzewicz 2001); between 100 and 200 in *Dysmicoccus newsteadi* (Pseudococcidae), *Kermes quercus* (Kermesidae), *Eriococcus buxi* (Eriococcidae), *Gossyparia spuria* (Eriococcidae), *Cryptococcus fagisuga* (Cryptococcidae), *Pseudochermes fraxini* (Cryptococcidae) (Szklarzewicz 1998a) and approximately 300 in *Porphyrophora polonica* (Margarodidae) (Szklarzewicz 1998b).

The ovarioles in *D. coccus* were observed at different stages of maturity (Figure 1A), which indicates that the maturing process of their ovaries occurs asynchronously. This asynchrony allows the females of this species to oviposit continuously during several days, even when separated from the host plant. The same asynchrony in ovarian maturity is also seen in other Coccoidea, such as in G. *spuria* (Szklarzewicz 1998a; Koteja et al. 2003). However, in *P. polonica* (Szklarzewicz 1998b; Koteja et al. 2003) and *Steingelia gorodetskia* (Steingeliidae) (Koteja et al. 2003) the ovaries develop synchronously; all ovarioles mature at the same time. According to Koteja et al. (2003) the Coccoidea groups with asynchronous ovary development have a prolonged oviposition period, whereas in species with synchronous ovary development this period is shorter.

Llanderal and Nieto (2001) mention that one female *D. coccus* deposits about 100 eggs. Thus, comparing the number of ovarioles present in *D. coccus* with the number of eggs the female can deposit, it may be deduced that the fertility of *D. coccus* is very low.

The ovarioles in D. coccus ovaries emerge radially all along the lateral oviducts. They lack a terminal filament, but in each of them it was possible to distinguish the germarium, the vitellarium, and a short pedicel. The lateral oviducts came out into the common oviduct, which finally communicates with the vagina (Figure 1A).

### Ovariole Structure

#### Germarium

The germarium is more or less spherical in shape (Figure 1A) and surrounded by an epithelial layer of very flat cells (Figure 1B and 1C). This germarium contained between seven and eight germinal cells, six or seven of which correspond to the nurse cells and one to the oocyte (Figure 1D). This contrasts with the large number of nurse cells and oocytes present in the germarium of other Coccoidea studied. For example, in *G.* *spuria* there are between three and seven nurse cells and one to four oocytes (Szklarzewicz 1998a), in *P. polonica,* there are between seven and fourteen nurse cells and four to six oocytes (Szklarzewicz 1998b), and in *S. gorodetskia* between 15 and 35 nurse cells and four to six oocytes are found (Koteja et al. 2003). Since it is considered that the number of germ cells in advanced Coccoidea is reduced and usually does not exceed 8 (Szklarzewicz 1998c), *D. coccus* belongs to the advanced Coccoidea.

The nurse cells in *D. coccus* were quite large and their cytoplasm had a granular aspect. The nuclei of these cells were also very large and of irregular shape and occupied almost all the cell volume. Their heterochromatin was lobated, and one or more nucleoli of variable size could be observed. The central part of the germarium (Figures 1B and 1C) was occupied by the trophic core, made up of an acellular structure. The nurse cells were connected to the trophic core by trophic processes. The trophic core and the trophic processes had a fibrillar structure that ran lengthwise through them (Figures 1B and 1E). In the germarium of *D. coccus* it was not possible to observe oocytes in previtellogenesis that subsequently would enter vitellogenesis (Figure 1B and 1E); this same characteristic has been observed in the most advanced Coccoidea. This characteristic is shared with *Quadraspidiotus ostraeformis* (Curtis) (Diaspididae), where it was also not possible to find previtellogenic oocytes in the germarium (Koteja et al. 2003). At the base of the germarium some prefollicular cells of approximately elongated form were distinguished (Figure 1E).

#### Vitellarium

The follicular cells had varying shapes including cylindrical, cubical and flat, depending on the degree of ovariole maturity. Their nuclei were almost spherical and centrally located; their heterochromatin was observed as a small package positioned in the center (Figures 1E and 1F). In the vitellarium there was always only one oocyte in vitellogenesis, which was connected to the germarium by the trophic cord during this phase (Figures 1B and 1E), contrasting with *S. gorodetskia,* in which from two to four oocytes in vitellogenesis may be found in the vitellarium (Koteja et al. 2003). The oocyte had a fairly large nucleus, whose position varied from central to completely eccentric (Figure 1F). Its heterochromatin was observed in the form of thin threads, among which several nucleoli could be perceived. The yolk of the oocytes in vitellogenesis appeared principally in the form of globules (Figure 1F). The trophic cord was also seen with a fibrillar structure, and these fibers even came to penetrate the top of the oocyte (Figure 1E).

#### Pedicel

This structure is a tube, basically shaped of a simple cubical epithelium with small nuclei, situated in the center. It was not possible to observe any specialization of these cells, which suggests the absence of glandular function or functions of any other type (Figure 1G).
